# Trends over two decades in life expectancy with complex health problems among older Swedes: implications for the provision of integrated health care and social care

**DOI:** 10.1186/s12889-022-13099-8

**Published:** 2022-04-14

**Authors:** Bettina Meinow, Peng Li, Domantas Jasilionis, Anna Oksuzyan, Louise Sundberg, Susanne Kelfve, Jonas W. Wastesson

**Affiliations:** 1 Aging Research Center, Department of Neurobiology, Care Sciences and Society, Karolinska Institutet and Stockholm University, Solna, Sweden; 2 Stockholm Gerontology Research Center, Stockholm, Sweden; 3 Max Planck Institute for Demographic Research, Rostock, Germany; 4 Chair of Demography and Health, School of Public Health, Bielefeld University, Bielefeld, Germany; 5 Division of Ageing and Social Change, Department of Culture and Society, Linköping University, Linköping, Sweden; 6 Department of Medical Epidemiology and Biostatistics, Karolinska Institutet, Solna, Sweden

**Keywords:** Sullivan method, Health expectancies, Complex health problems, Multimorbidity, Multi-domain health measures, Integrated care

## Abstract

**Background:**

Due to population aging, it is essential to examine to what extent rises in life expectancy (LE) consist of healthy or unhealthy years. Most health expectancy studies have been based on single health measures and have shown divergent trends. We used a multi-domain indicator, complex health problems (CHP), indicative of the need for integrated medical and social care, to investigate how LE with and without CHP developed in Sweden between 1992 and 2011. We also addressed whether individuals with CHP more commonly lived in the community in 2011 compared to earlier years.

**Methods:**

CHP were defined as having severe problems in at least two of three health domains related to the need for medical and/or social care: symptoms/diseases, cognition/communication, and mobility. The Swedish Panel Study of Living Conditions of the Oldest Old (SWEOLD), a nationally representative survey of the Swedish population aged ≥ 77 years with waves in 1992, 2002 and 2011 (n≈2000), was used to estimate the prevalence of CHP. Age- and gender-specific death rates were obtained from the Human Mortality Database. The Sullivan method was deployed to calculate the remaining life expectancy with and without CHP. The estimates were decomposed to calculate the contribution of changes from morbidity and mortality to the overall trends in LE without CHP.

**Results:**

Between 1992 and 2011, both total LE (+ 1.69 years [95% CI 1.56;1.83] and LE without CHP (+ 0.84 years [-0,87;2.55]) at age 77 increased for men, whereas LE at age 77 increased for women (+ 1.33 [1.21;1.47]) but not LE without CHP (-0.06 years [-1.39;1.26]). When decomposing the trend, we found that the increase in LE with CHP was mainly driven by an increase in the prevalence of CHP. Among individuals with CHP the proportion residing in care homes was lower in 2011 (37%) compared to 2002 (58%) and 1992 (53%).

**Conclusions:**

The findings, that an increasing number of older people are expected to live more years with CHP, and increasingly live in the community, point towards a challenge for individuals and families, as well as for society in financing and organizing coordinated and coherent medical and social services.

**Supplementary Information:**

The online version contains supplementary material available at 10.1186/s12889-022-13099-8.

## Background

Life expectancy is increasing internationally and most deaths in low-mortality countries now occur around the ages of 85 to 90 years [1] and are seldom sudden [2, 3]. Deaths after age 80 are often preceded by a period with chronic conditions, functional loss, and dependency [2, 4–6]. For example, many age-related conditions are not curable but can be managed for years [5, 7, 8]. The extent to which longer lives are accompanied by a compressed, postponed, or expanded period with health problems and care needs has broad ramifications for individuals and families, as well as for financing and organizing appropriate health care and social care.

In international comparison, Sweden has a comprehensive and universal system for health- and social care for older people, primarily financed by local taxes. In the highly decentralized care system, the 21 regions provide, manage and finance health care and the 290 municipalities are responsible for social care. Home-care care and care homes are the main forms of social care for older people. Access to social care is needs-based (not means-tested), available to all inhabitants aged 65 years or older whose need of support with personal care and/or household chores is approved by municipal need assessors. For about half a century “*Aging in place”*, i.e., supporting older people in their own homes, instead of in care homes, has been a key and guiding strategy in addressing older adults’ care needs. The policy has been embraced by older people and their families, as well as policy makers [9]. Yet, during the past two decades, economic constraints in many municipalities have been challenging the financial sustainability of the Swedish long-term care system. This has contributed to the further strengthening of the *“aging in place”* policy, involving a drastic reduction of beds in care homes, as well as of hospital beds [9]. Providing up to ~ 120 h home care per month is a more cost-effective alternative for the municipality compared to the costs for a bed in a care home. Most people enter the social care system through home care and a move to a care home is only approved if more intense home would still be insufficient. In recent years, the downsizing of care homes entailed a shorter length of stay, with an increase in the proportion of people who moved in shortly before death [10, 11].

If older people survive longer with extensive and complex care needs, and if they also to a greater extent remain in their own homes, despite such comprehensive and complex care needs, this development will reinforce the necessity and challenges of the provision of well-coordinated and coherent health care and social care in the community.

To address the question to what extent increased life expectancy entails healthy and unhealthy years, the concept of health expectancies has been developed and estimates the interaction of mortality and morbidity [12]. Divergent time trends in health expectancies have been reported across countries, subgroups of populations (e.g., regarding gender, age, and ethnic groups), time periods and the health indicator used [13]. The Global Burden of Disease Study 2016 provided healthy life expectancy estimates for 195 countries and territories between 1990 and 2016 [13]. At a global level, healthy life expectancy continued to show improvements, although many populations were facing an increase in time with functional limitations [13]. Most health expectancy studies that have suggested a trend towards shorter time spent with poor health at the end of life – a compression of morbidity – have used health indicators based on disability (e.g., activities of daily living, ADLs) [14–18] or cognitive impairment [19–22]. This trend has been attributed to decreasing rates of disability among more recent cohorts of older people, especially those under the age of 85 [14, 23, 24].

In contrast, health expectancy studies finding support for an expansion of morbidity during the last two decades have frequently focused on chronic diseases/conditions [14, 22, 25–27]. That more people are getting diagnosed at an earlier stage in later cohorts has probably contributed to this development.

However, while trends in health expectancies for specific single health indicators have developed differently, health problems often exist simultaneously and are interrelated in old age [28, 29]. Hence, in this study we use a composite health measure including both chronic diseases/conditions and functional limitations to address the remaining lifetime with and without combined medical conditions and functional limitations. Such composite health measures may to a larger extent reflect the broader impact of health problems experienced by individuals and their families [29]. From a policy and planning perspective, such indicators can be useful to assess, on population level, the need for integrated health- and social care to meet multiple complex care needs [30].

Yet, only a few studies have analyzed years lived with multiple morbid conditions and most of them focus on multimorbidity (the presence of two or more diseases) or frailty (a state that is characterized by cumulative decline in many physiological systems, vulnerability to physiologic stressors and risk of adverse outcomes) [31, 32]. Both concepts rely primarily on clinical data, such as diagnosed diseases and physiological markers. Therefore, they may indicate a need for medical care or a risk for *subsequent* disability and associated social care needs. Yet, neither multimorbidity nor frailty capture *actual* needs for combined health care and social care [29]. One exception is a study based on the British Cognitive Function and Ageing Studies that integrated geriatric conditions and dependency in activities of daily living (ADL) into a measure of dependency [33]. Results showed that years lived with dependency from age 65 increased between 1991 and 2011 [33].

Based on representative samples of the Swedish population aged 77 or older, we have previously developed a composite measure of complex health problems (CHP) that reflects the simultaneous presence of medical conditions, and physical and cognitive functional limitations. This measure may serve as a crude indicator of the proportion of older adults who need both health- and social care, but who have limited capacity to navigate a fragmented care system and to demand and coordinate care from different providers [29, 34]. The presence of CHP has also been shown to be highly predictive of 4-year mortality, confirming that the measure captures a very vulnerable group of older adults [35]. The objective of this study was to investigate how life expectancy with and without CHP has developed between 1992 and 2011 in Swedish men and women aged 77 or older. We also addressed whether it has become more common among individuals with CHP to live in the community, instead of in care homes, which would increase the need of well-coordinated and coherent health- and social care in the community.

## Methods

### Data

Prevalence of CHP was based on three waves (1992, 2002 and 2011) of the Swedish Panel Study of Living Conditions of the Oldest Old (SWEOLD), which covers representative samples of the Swedish population aged 77 or older (*n* = 2016) [36]. The SWEOLD samples are nationally representative with respect to the distribution of age, gender, geography, and proportion of people living in care homes. Age- and sex- specific death rates for the survey years were obtained from the Human Mortality Database [37].

In 2011, 85- to 99-year-olds were oversampled to increase the precision of measurements in the oldest age groups. Sample weights have been applied to adjust for this oversampling [36]. Response rates across the survey waves were high (between 86 and 90%) due to intensive fieldwork that was designed to include very frail and cognitively impaired older persons. Whenever possible, direct face-to-face interviews were conducted in the respondent’s dwelling. To avoid the loss of the most frail and impaired individuals in the sample, proxy interviews were conducted with a close family member if the respondent, due to cognitive or physical impairment felt unable to participate.

Data collection and analyses for the three survey waves have been approved by the Uppsala University Hospital Ethical Committee (1992; Dnr 247/91), the Karolinska Institutet Ethical Research Committee (2002; Dnr 03–413), and the Regional Ethical Review Board in Stockholm (2011; Dnr 2010/403–31/4). No further administrative permissions were required to access and use the data. Informed consent was obtained from all respondents who were able to give it. For cognitively/physically impaired respondents who were unable to give informed consent and to answer the survey questions themselves, a close family member or legally authorized representative were consulted and given the possibility to refuse participation or to provide consent to conduct a proxy interview.

### Health dimensions and indicators

The measure of CHP was based on three health domains that are related to the need for different types of care services: symptoms/diseases, cognition/communication, and mobility [34, 38]. Typically, symptoms and diseases imply need for medical care. Functional limitations, such as mobility problems are mainly handled by social services and/or informal caregivers. Serious limitations in cognitive/communication skills often require help from both, health care and social care providers. Persons with serious problems in at least two of the three health domains were considered to have CHP [34].

To provide comparable and nationally representative estimates, we used measures that were available for the complete sample, including proxy interviews. This excluded subjective evaluations of health and tests of function (except for the cognition test, see below). Severe problems in each of the health domains were identified as briefly described below. Detailed information on the items included in each health domain, as well as proportions of respondents with limitations in each of the health items, can be found in Table 1 in the Supplementary file.

**Table 1:** Sample characteristics and prevalence of severe problems in three health domains 1992, 2002 and 2011

	**1992** *n*** = 537**	**2002** *n*** = 621**	**2011**^a^ *n*** = 831**	***p*-value**^b^** 1992–2002**	***p*-value**^b^** 1992–2011**
Proxy interview	11.7	13.2	17.8	0.414	0.003
Females	60.8	59.3	62.6	0.581	0.528
Institutional care	18.6	22.5	11.7	0.088	0.003
Age range	77–98	77–99	77–101		
Mean/median age	82.4/82.0	83.3/83.0	83.4/83.0	0.001^c^	< 0.001^c^
Multiple severe symptoms/diseases	20.5	32.3	34.2	< 0.001	< 0.001
Severe mobility limitations	19.5	26.0	24.4	0.008	0.048
Poor cognition/ Communication	25.7	33.3	29.8	0.003	0.116
No serious problems in any domain	56.4	41.7	45.1	< 0.001	< 0.001
Serious problems in one domain	25.5	32.1	29.3	0.011	0.146
Serious problems in 2–3 domains	18.1	26.1	25.6	0.001	0.002

Between 2002 and 2011, the prevalence of severe problems did not change significantly in any of the health domains

^a^ Percentages were weighted for an oversampling (*n* = 257) of persons aged 85 years or older in the 2011 survey

^b^ based on chi2 test

^c^ *p*-value refers to t-test of mean differences. Data: The Swedish Panel Study of Living Conditions of the Oldest Old 1992, 2002, 2011. Own calculations

#### Symptoms/diseases

Respondents were asked whether they have had any of 14 common symptoms and diseases during the previous 12 months (see Supplementary file for a list). For each item, mild problems were coded as 1 and severe problems were coded as 3. This resulted in a summed index that ranged from 0 to 42. A cut-off for severe problems for this domain was set at the highest quintile for the 1992 sample (corresponding to 9 on the summed scale) [34]. Accordingly, persons belonging to the highest quintile had, e.g., at least three severe symptoms/diseases or two severe and three mild. The same cut-off was used for the survey waves in 2002 and 2011.

#### Mobility

This domain was an index of four items: the reported ability to walk 100 m fairly briskly without difficulties, walk up and down stairs, rise from a chair without difficulty, and stand without support. Limitations in at least three of the four activities were coded as serious mobility problems.

#### Cognition/communication

Cognition was assessed using an abridged version of the Mini-Mental-State Examination [39] that has been validated against the full MMSE scale and clinical dementia diagnoses [40]. Nearly all proxy-interviews were due to cognitive problems (in a few cases aphasia) according to interviewer notes. Therefore, respondents who could not be interviewed directly, scored below the cutoff (< 7 out of 11) in the MMSE test, or refused to do the test were coded as having serious cognitive/communication problems. Thus, this measure is not only an indicator of cognition but rather captures respondents with cognitive impairment and/or who were unable communicate with the interviewer. Cognition/communications problems entail an important dimension of dependence in daily life, not least indicating need for help with navigating the health and social care system and managing one’s own care contacts.

#### Use of social care

The interviewer noted whether an individual lived in a care home or not. Respondents were asked whether they received home care or not.

### Statistical analyses

The Sullivan method was used to estimate life expectancy at age 77 with and without CHP for women and men separately [41]. Age-, gender- and period-specific prevalence of CHP was combined with standard period life tables. Age-specific prevalence of CHP was smoothed using a logistic regression model with linear and quadratic terms of age [42]. Using a decomposition method by Andreev et al. [43], the changes in life expectancy with and without CHP between the survey years were decomposed into the contributions a) attributable to mortality change and b) the contributions attributable to changes in the prevalence of CHP. Microsoft Excel and R (version 3.5) were used for life table analysis, smoothing, and decomposition analysis. STATA SE15 was used to obtain prevalence of health problems, tests for statistical significance and 95% confidence intervals (95% CI). All statistical tests were two-sided; the differences in prevalence between the selected time points were statistically significant if *p* < 0.05.

## Results

The proportion of people with severe problems increased in each of the three health domains between 1992 and 2011 among Swedes aged 77 or older (Table 1). The most distinct increase, from 20.5% percent to 34.2% (*p* < 0.001), occurred for the severe symptoms/diseases domain. Severe mobility limitations increased from 19.5% to 24.4% (*p* < 0.05). The slight increase of severe cognition/communication problems from 25.7% to 29.8% was not statistically significant (*p* = 0.116). The proportion of the population with CHP, i.e., severe problems in two or three health domains, increased by 41%, from 18.2% in 1992 to 25.6% in 2011 (*p* < 0.01). As for the three health domains separately, the prevalence of CHP increased mainly during the first decade of follow-up, i.e., between 1992 and 2002, and remained stable between 2002 and 2011. Patterns were similar for men and women, although women generally had more health problems (not shown).

### Use of social care

**Fig. 1:**
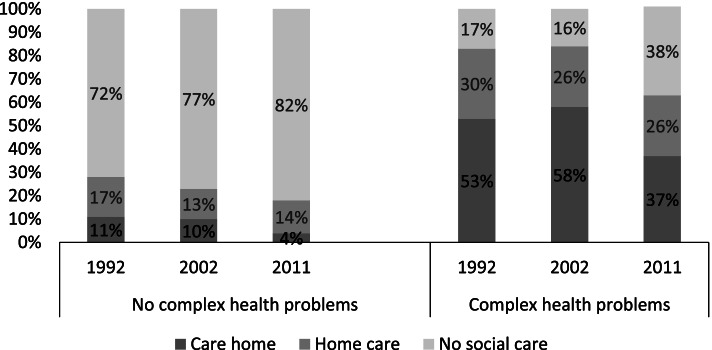
Long-term care use among individuals with and without complex health problems, 1992–2011. The bars show the proportions of individuals living in a care home, using home care, or no social care, stratified according to the presence of complex health problems or not. Data: The Swedish Panel Study of Living Conditions of the Oldest Old 1992, 2002, 2011. Own calculations

Figure 1 shows the use of social care (home care or care home) for individuals with CHP compared with those without CHP. Between 1992 and 2002, the proportion of individuals with CHP who lived in a care home increased slightly from 53 to 58%, whereas the proportion who used home care slightly decreased. During the following decade, among individuals with CHP, the proportion living in a care home care dropped to 37%, while the proportion with home care in the same group remained constant. The proportion of individuals with CHP who did not use any social care services more than doubled, from 16 to 38%. Few individuals *without* CHP lived in a care home.

### Life expectancy with and without complex health problems

Between 1992 and 2011, remaining life expectancy at age 77 increased by 1.69 years for men (from 8.08 to 9.77 years) and by 1.33 years for women (from 10.38 years to 11.71 years). For both genders and at all time points, most of the remaining life expectancy consisted of years without CHP (Table 2).

**Table 2:** Remaining life expectancy at age 77 and estimated remaining life expectancy with and without complex health problems, by gender and survey year

	**1992**	**2002**	**2011**	**Diff 2011–1992**
**Men**				
LE	8.08 (8.01–8.14)	8.78 (8.72–8.84)	9.77 (9.70–9.84)	1.69
LE without CHP Years (95% CI)	6.78 (5.83–7.73)	6.76 (5.94–7.57)	7.62 (6.86–8.38)	0.84
LE with CHP Years (95% CI)	1.30 (1.10–1.50)	2.02 (1.75–2.29)	2.15 (1.96–2.35)	0.85
**Women**				
LE	10.38 (10.31–10.44)	10.78 (10.72–10.84)	11.71 (11.65–11.78)	1.33
LE without CHP Years (95% CI)	7.77 (7.03–8.51)	7.13 (6.58–7.68)	7.71 (7.12–8.29)	-0.06
LE with CHP Years (95% CI)	2.61 (2.29–2.93)	3.65 (3.31–3.99)	4.01 (3.69–4.33)	1.40

Data: The Swedish Panel Study of Living Conditions of the Oldest Old 1992, 2002, 2011. Human Mortality Database. Own calculations. Abbreviations: *LE* life expectancy, *CI* confidence interval

For men, half of the additional expected life years (0.85 years) consisted of years with CHP. While the increase of years with CHP mainly occurred during the first half of the period (1992–2002), years without CHP (0.84) increased essentially during the second half (2002–2011).

Among women, the entire increase of expected life years (1.33) consisted of years with CHP. Life expectancy without CHP decreased by 0.64 years between 1992 and 2002 and then increased again by 0.58 years between 2002 and 2011, resulting in almost no change over the 19-year period. This resulted in an increase of years with CHP for men and women, and more so for women, who could expect to live with CHP about twice as long as men at all three time points.

**Fig. 2:**
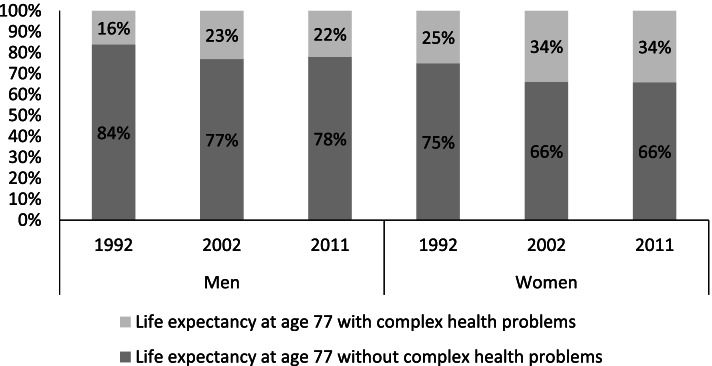
Estimated proportions of remaining life expectancy at age 77 to be lived with and without complex health problems 1992–2011, by gender. The bars show estimated proportions of remaining life years to be lived with and without complex health problems for the years 1992, 2002 and 2011, for men and women. Data: The Swedish Panel Study of Living Conditions of the Oldest Old 1992, 2002, 2011, Human Mortality Database. Own calculations

Figure 2 illustrates, that in relation to total life expectancy at age 77, the proportion of time lived with CHP increased in both men (from 16 to 23%) and women (from 25 to 34%) between 1992 and 2002 and remained unchanged thereafter.

### Decomposition of changes in life expectancy with and without complex health problems into mortality and morbidity effects

Among men, between 1992 and 2011, the increase in life expectancy without CHP (0.84 years) was mainly driven by a mortality effect (1.27 years), which was partially offset by the effect of increased prevalence of CPH (-0.43 years) (Table 3). For women, years without CHP decreased somewhat during the same period (0.06 years) because the positive mortality effect (0.79 years) was completely offset by a slightly greater negative morbidity effect (-0.85 years), i.e., an increase in the prevalence of CHP (Table 3).

**Table 3:** Contribution of the effects of mortality and prevalence of complex health problems to changes in life expectancy without complex health problems, 1992–2011

	**Men**			**Women**		
	**1992–2002**	**2002–2011**	**1992–2011**	**1992–2002**	**2002–2011**	**1992–2011**
**Change in LE**	0,70	0,99	1,69	0,40	0,93	1,33
**Change in LE without CHP**	-0.02	0.86	0.84	-0.64	0.58	-0.06
**Mortality effect**	0.51	0.66	1.27	0.26	0.47	0.79
**CHP-effect**	-0.53	0.20	-0.43	-0.89	0.11	-0.85

Data: The Swedish Panel Study of Living Conditions of the Oldest Old 1992, 2002, 2011, Human Mortality Database. Own calculations. Abbreviations: *LE* life expectancy, *CHP* complex health problems

During the first part of the period (1992 and 2002), the opposite contributions of mortality effect (0.51 years) and effect of CPH prevalence (-0.53 years) to increasing LE with CPH were similar among men. Among women, the contribution of effects of CPH prevalence (-0.89 years) was much larger than that of mortality effects (0.26 years). Between 2002 and 2011, on the other hand, for both sexes the increase of life expectancy without CHP was predominantly driven by mortality effects.

## Discussion

In this nationally representative study of older adults in Sweden, we found that life expectancy at age 77 continuously increased between 1992 and 2011. Overall, the number of years lived with CHP and the share of life with CHP increased for both men and women. In 2011, from age 77, men spent on average 2.15 years and women 4.01 years with CHP. For women, the added life years consisted only of years with CHP, while the added years for men were equally distributed between years with and without CHP. Fewer individuals with CHP resided in a care home in 2011 compared to one and two decades before.

The composite measure of CHP captures individuals with severe problems in several health domains, including both chronic diseases/symptoms and functional limitations, and is therefore highly indicative of the need for both health- and social care, usually involving several providers. Yet, older adults with CHP are also likely to have a limited capacity to navigate the care system and to demand and coordinate care from different providers.

The finding that there was no sign of a decrease in the time spent with CHP, has important implications for resource needs and funding of care services, and highlights the importance of the development and improvement of well-coordinated and integrated health care and social care. This is of particular importance, given our results that an increasing number and proportion of older adults with CHP lives in the community, and not in care homes.

### Increase of life expectancy with complex health problems in context of the literature

The trend of an increase of years lived with CHP can mainly be attributed to an increasing prevalence of CHP in the first part of the period (1992–2002), which all three health dimensions contributed to. Most previous studies on time trends in health expectancies have used single health indicators and divergent trends have been shown for different indicators, countries and time-points compared [21]. Given limits in comparability, our results of an increase in years lived with complex health problems point towards a similar trend as a few previous studies that have used composite health measures. For example, expected life years spent with cognitive impairment and disability combined increased slightly in Hong Kong between 2001/02 and 2011/12 [19]. A study based on the British Cognitive Function and Ageing Studies integrated dependency in activities of daily living and geriatric conditions into a measure of dependency. Results showed a significant increase of years lived from age 65 with both low dependency (requiring help less often than daily) and high dependency (24-h care) between 1991 and 2011 [33]. As in the present study, the development was more pronounced for women than for men [33]. On the other hand, results from the Longitudinal Aging Study Amsterdam reported an increase in years lived with combined multimorbidity and disability between 1993 and 2016 for men, but no clear trend for women [22]. Studies focusing on diseases and/or physiological indicators found an increase in time spent with six or more chronic conditions and polypharmacy between 2005 and 2014 in Germany, both in absolute years and as an increasing proportion of life [32].

A study among community-dwelling people conducted in 2011 revealed substantial differences in life expectancy with frailty at age 70 across 15 European countries, with estimates ranging between 0.1 to 1.8 years for men and from 0.4 to 5.5 years for women [31]. However, to our knowledge, there is a lack of studies on time trends in life expectancy with frailty.

That women in our study at all three time points could expect to live with CHP twice as long as men reflects the male–female health-survival paradox [44], that has been observed for most other single as well as composite health measures, such as multimorbidity [32, 45], frailty [31, 46], disability [33] as well as poor physical and cognitive health combined [19, 22].

Our decomposition analyses indicated that the increase in expected life years with complex health problems among women in the period of 1992–2002 was mainly driven by change in the prevalence of CHP, rather than mortality, while the contributions of these forces were similar among men. The increase of years without CHP, on the other hand, was predominantly driven by change in mortality, rather than the prevalence complex health problems, especially during the latter part of the period (2002–2011), and among men.

Increased prevalence of diseases and chronic conditions, as well as some functional limitations, have also been widely reported in Sweden [47] and internationally [14, 25, 33], although evidence on trends in the prevalence of disability seems more favorable [47]. Earlier diagnoses, successful life-saving interventions and better disease-control probably contribute to an increase of the prevalence of chronic conditions and functional limitations. Improved survival with disease may also entail an increased risk for further diseases [48].

### Strengths and limitations

Some strengths and limitations of this study must be acknowledged when interpreting the results. Although the sample sizes of the three SWEOLD waves are relatively small, a main strength of the study is that prevalence estimates can be assumed to be highly representative. All survey waves comprise nationally representative samples of the population aged 77 or older with high response rates (> 85%), including people with poor cognition and those living in a care home. Identical study design and methods were used over the decades. To avoid non-response, and thereby an underestimation of health problems on population level, proxy interviews with a close family were conducted for those individuals who were too frail and/or cognitively impaired to participate by themselves [36].

The health indicators are mostly self-reported (except the cognitive test), which could introduce bias in the health assessments. In national population surveys, for practical reasons, clinical precision cannot be accomplished. However, crude health measures that are not clinically relevant, but allow the inclusion of proxy interviews, can be highly suitable for national surveys aimed at informing decision makers for policy and planning.

To cover the most vulnerable subset of the older population, we made our definition of complex health problems restrictive, setting high thresholds for severe problems within each domain. However, people with severe problems in only one domain could also need care from a variety of providers. Thus, rates of CHP as measured in this study, could to some extent underestimate actual needs for integrated health care and social care in the older population.

Moreover, the Sullivan method, which we used to calculate health expectancies, does not take transitions between health states into account, which could potentially introduce bias in the health estimates [41]. However, recovery from complex health problems, involving severe problems in several health domains, is likely to be limited in high age, and the estimates based on the Sullivan method have proven reliable, as long as prevalence is regular and smooth over time [49, 50].

### Implications for social policy

Our results, that more people live longer with CHP, and increasingly remain in their own homes, has important implications against the background of recent developments in social policy. To some extent, the non-linear trend, showing that the increase in the prevalence of CHP and expected live years with CHP mainly occurred in the first ten years of the study period (1992–2002) with little change thereafter (2002–2011), may be seen as encouraging for the coming decades. However, even under the assumption that the prevalence of CHP continued to remain unchanged after 2011, the *number* of individuals aged 77 or older with CHP in Sweden can be expected to have risen from around 172,000 in 2011 to 202,000 in 2020 (based on demographic statistics from Statistics Sweden) [37]. An additional 40% increase to 280,000 individuals with CHP could be expected by 2030, when the large cohorts born in the 1940s will reach 80 to 90 years of age, still assuming unchanged prevalence of CHP since 2011 [37].

Sweden was a forerunner in providing formal social care, especially home-based care. Even before aging populations were an international concern, the comprehensive needs-based social care system expanded rapidly as part of the general expansion of the welfare state during the 1960s and 1970s. The principle of “aging in place”, that is, to support older people in their own home, became a political ambition as early as in the mid-1950s, when home care was introduced after an intense public debate about poor conditions in former old-age-homes [51]. During the following three decades, the number of home care recipients increased at a faster pace than the population aged ≥ 80 years and the number of care home residents. The expansion of home-based care, giving older people the option to remain longer in their own homes, was embraced by older people themselves, their families, as well as policymakers [9, 51].

However, since the economic recession in the early 1990’s, in parallel with an increase in the number and proportion of older adults with CHP, focus has been on cost containment. This has entailed substantial cutbacks to sustain the publicly financed system and the further strengthening of the home-oriented approach, rather than care homes, has been the most salient strategy for system adaption to contain costs [9]. Accordingly, since 2000, 30 percent (37,000) of the 121,000 beds in care homes have been cut down, currently reaching 13% of people aged ≥ 80 years [52]. Yet, the downsizing of care homes during the past two decades has not been compensated by a corresponding increase of the proportion of individuals receiving home care (around 21–23% aged ≥ 80 years) and particularly not in terms of the intensity of care. Also, half the hospital beds have been cut since the 1990s [9]. Fewer beds in care homes and shorter hospital stays have entailed that very frail old people increasingly are cared for at home [9]. Our results confirmed that a substantially higher proportion of those with CHP lived in the community 2011 compared to previous decades. Among older adults *without* CHP, very few individuals lived in a care home, indicating that this most comprehensive form of social care is concentrated to those with the greatest care needs. Since another 10,000 beds in care homes have been cut down since 2011, the trend that older people live at home, despite substantial and complex care needs, has continued during the past years. This development has led to a public debate, to what extend higher eligibility thresholds for care homes have contributed to a situation where home care for some older people may imply a lack of attractive options [9].

The decline in formal care for older people has also been accompanied by a *refamiliasation* of care [53]. In our results, this could potentially be reflected in the increasing proportion of persons with CHP who reported no use of formal social care. Although in international comparison formal care provision is extensive in Sweden, informal carers have been estimated to provide approximately two thirds of all care for older people living at home and their role is increasing [9, 53]. It has also been shown that caring for older parents has negative repercussions on middle-aged children’s ability to work to the extent they would prefer [54].

Another development in Swedish eldercare, particularly affecting the situation of older people with CHP who live at home, is the marketization of care [9, 54]. Despite municipalities being responsible for the funding of social care, New Public Management inspired reforms since the 1990s involved the free establishment of private companies providing publicly funded care. Subsequently, this has led to a large number of independent formal care providers, especially in bigger cities. This has yielded an additional layer of complexity in navigating the care system.

While our results show that older people increasingly live in the community despite CHP, the ability to navigate the health- and social care system, and to manage and coordinate care contacts with different providers, generally decreases with poor health [55]. This is particularly challenging in a fragmented system, where health care is financed and organized at the regional level while municipalities are responsible for social care [56]. The highly decentralized Swedish care system, as well as the large number of independent care providers, who for example may have different journal systems, has been reported to result in suboptimal care for individuals who need a variety of both health- and social care services [56].

Thus, in conclusion, our results, that more people live longer with CHP, and increasingly remain in their own homes, reinforce the importance of developing new models of communication and coordination between providers to provide coherent health- and social care for the most vulnerable individuals living in the community.

## Supplementary Information

**Additional file 1:** **Supplementary Table 1:** Prevalence of health problems included in three health domains (symptoms/diseases, mobility limitations, cognitive/communication problems) and severe health problems in each health domain in 1992, 2002 and 2011.

## Data Availability

The datasets used and/or analyzed during the current study are available from the corresponding author on reasonable request. Public access to the database may be given if applicants have a scientific affiliation, sign a statement that the data will only be used for scientific purposes, and that the scientific project have ethical approval. Applicable sections of The Swedish Research Council (VR) principles for conducting research in humanities and the social sciences must be adhered to (http://www.codex.vr.se/en/forskninghumsam.shtml). Data will be available after the above-mentioned documents have been received, by email at the SWEOLD Research Data Center dataaccess@sweold.se More information can be found at www.sweold.se.

## Abbreviations

SWEOLD: The Swedish Panel Study of Living Conditions of the Oldest Old
LE: Life expectancy
CHP: Complex health problems
CI: Confidence interval
