# Supplementary material for: Trends over two decades in life expectancy with complex health problems among older Swedes: implications for the provision of integrated health care and social care
Source: BMC Public Health. 2022 Apr 14;22:759. doi: 10.1186/s12889-022-13099-8 (PMC9012017; doi:10.1186/s12889-022-13099-8)
Supplement: Supplementary file 1 — Additional file 1: Supplementary Table 1: Prevalence of health problems included in three health domains (symptoms/diseases, mobility limitations, cognitive/communication problems) and severe health problems in each health domain in 1992, 2002 and 2011. [file 12889_2022_13099_MOESM1_ESM.docx]

**Supplemental Materials:**

**Trends over two decades in life expectancy with complex health problems among older Swedes: implications for the provision of integrated healthcare and social care**

Bettina Meinow^[[1]](#footnote-1),^^[[2]](#footnote-2)^  Peng Li^[[3]](#footnote-3)^  Domantas Jasilionis^3^ Anna Oksuzyan^3,^^[[4]](#footnote-4)^ Louise Sundberg^1^

Susanne Kelfve^[[5]](#footnote-5),1^ Jonas W Wastesson^[[6]](#footnote-6),1^

**Detailed information on the items for each health domain that is included in the measure of complex health problems (CHP).**

Supplementary Table 1: Prevalence of health problems included in three health domains (symptoms/diseases, mobility limitations, cognitive/communication problems) and severe health problems in each health domain in 1992, 2002 and 2011

|  |  | | **1992**  **n=537**  **(%)** | **2002**  **n=621**  **(%)** | ***p*-value for change 1992-2002** | **2011^a^**  **n=831**  **(%)** | ***p*-value^b^ for change 2002-2011** | ***p*-value^b^ for change 1992-2011** |
| --- | --- | --- | --- | --- | --- | --- | --- | --- |
| **Symptoms/diseases^c^** | | |  |  |  |  |  |  |
| General fatigue/  sleeplessness | Mild | | 37.2 | 43.3 | 0.050 | 44.4 | 0.713 | 0.026 |
|  | Severe | | 15.2 | 22.1 | 0.006 | 24.1 | 0.389 | <0.001 |
| Dizziness | Mild | | 27.9 | 30.9 | 0.295 | 33.3 | 0.370 | 0.065 |
|  | Severe | | 5.2 | 8.4 | 0.089 | 7.2 | 0.452 | 0.246 |
| Leg ulcers | Mild | | 2.2 | 4.7 | 0.150 | 2.7 | 0.056 | 0.732 |
|  | Severe | | 1.2 | 2.3 | 0.161 | 2.1 | 0.878 | 0.200 |
| Diabetes | Mild | | 8.1 | 6.9 | 0.62 | 13.2 | <0.001 | 0.032 |
|  | Severe | | 3.4 | 3.2 | 0.907 | 5.3 | 0.068 | 0.231 |
| Stomachache | Mild | | 12.2 | 17.7 | 0.008 | 16.1 | 0.429 | 0.056 |
|  | Severe | | 4.9 | 5.3 | 0.745 | 4.2 | 0.327 | 0.526 |
| Myocardial infarction/other heart problems | Mild | | 16.4 | 15.8 | 0.792 | 23.5 | 0.001 | 0.005 |
|  | Severe | | 3.9 | 7.6 | 0.007 | 8.3 | 0.615 | 0.002 |
| Stroke | Mild | | 0.7 | 3.7 | 0.002 | 4.1 | 0.733 | 0.001 |
|  | Severe | | 2.5 | 2.6 | 0.963 | 1.5 | 0.192 | 0.214 |
| Breathlessness | Mild | | 26.0 | 26.1 | 0.981 | 25.5 | 0.805 | 0.875 |
|  | Severe | | 6.8 | 7.6 | 0.643 | 7.9 | 0.809 | 0.507 |
| Chest pain | Mild | | 18.8 | 15.1 | 0.135 | 19.3 | 0.054 | 0.838 |
|  | Severe | | 7.9 | 8.9 | 0.655 | 4.6 | 0.002 | 0.044 |
| Hypertension | Mild | | 19.1 | 20.9 | 0.479 | 38.3 | 0.000 | <0.001 |
|  | Severe | | 2.7 | 6.8 | 0.001 | 8.0 | 0.402 | <0.001 |
| Joint pain | Mild | | 25.5 | 33.2 | 0.011 | 37.5 | 0.106 | <0.001 |
|  | Severe | | 15.2 | 23.4 | 0.001 | 25.0 | 0.509 | <0.001 |
| Back pain | Mild | | 29.6 | 27.7 | 0.547 | 29.5 | 0.489 | 0.975 |
|  | Severe | | 16.6 | 24.5 | 0.006 | 23.8 | 0.793 | 0.010 |
| Shoulder pain | Mild | | 24.5 | 30.3 | 0.051 | 27.4 | 0.258 | 0.317 |
|  | Severe | | 10.1 | 12.4 | 0.272 | 15.2 | 0.149 | 0.021 |
| Low BMI | Mild | | 23.0 | 24.8 | 0.505 | 17.6 | 0.002 | 0.031 |
|  | Severe | | 0.8 | 0.3 | 0.248 | 0.7 | 0.380 | 0.716 |
| **Multiple severe diseases/ symptoms^3^** |  | | **20.5** | **32.3** | **<0.001** | **34.2** | **0.470** | **<0.001** |
| **Mobility limitations^d^** |  | |  |  |  |  |  |  |
| Cannot walk 100 m fairly briskly without problems |  | | 37.8 | 49.8 | <0.001 | 41.2 | 0.002 | 0.283 |
| Cannot stand without support |  | | 12.1 | 15.6 | 0.111 | 12.4 | 0.094 | 0.871 |
| Cannot rise from a chair |  | | 23.0 | 33.3 | <0.001 | 32.0 | 0.632 | 0.001 |
| Cannot walk up stairs |  | | 38.2 | 43.7 | 0.081 | 47.1 | 0.232 | 0.005 |
| **Severe mobility limitations** |  | | 19.5 | 26.0 | 0.008 | 24.4 | 0.581 | 0.048 |
| **Cognition/communication^e^** | | |  |  |  |  |  |  |
| Low MMSE | |  | 14.0 | 20.1 | 0.006 | 12.0 | 0.003 | 0.978 |
| Proxy interview | |  | 11.7 | 13.2 | 0.414 | 17.8 | 0.020 | 0.003 |
| Poor cognition/ communication | |  | **25.7** | **33.3** | 0.003 | **29.8** | 0.176 | 0.116 |

Notes: ^a^ Percentages were weighted for an oversampling (n=257) of persons aged 85 years or older in the 2011 survey. ^b^ based on chi2 test. ^c^ “No” was coded as 0, “Yes, mild problems” was coded as 1 and “Yes, severe problems” was coded as 3. The summed diseases/symptoms domain ranged from 0 to 42. A cut-off (9) for multiple severe diseases/symptoms was determined at the highest quintile for the 1992 sample and the same cut-off was used for the 2002 and 2011 sample. ^d^ Persons not able to perform at least three of the four activities were coded as having serious mobility problems. ^e^ Persons who scored <7 on the cognitive test or who were not able to perform an interview (in almost all cases due to cognitive problems) were considered to have poor cognition/communication skills. All missing values were treated as incorrect answers. answer the questions. Missing values on each of the items were treated as incorrect answers (no correct answers on any on the MMSE items: 1992: n=13; 2002: n=16; 2011: n=3). Data: The Swedish Panel Study of Living Conditions of the Oldest Old 1992, 2002, 2011. Own calculations.

1. ^Aging Research Center, Department of Neurobiology, Care Sciences and Society,^ [↑](#footnote-ref-1)
2. ^Stockholm Gerontology Research Center, Stockholm, Sweden^ [↑](#footnote-ref-2)
3. ^Max Planck Institute for Demographic Research, Rostock, Germany^ [↑](#footnote-ref-3)
4. ^Chair of Demography and Health, School of Public Health, Bielefeld University, Bielefeld, Germany.^ [↑](#footnote-ref-4)
5. ^Department of Culture and Society, Linköping University, Linköping, Sweden^ [↑](#footnote-ref-5)
6. ^Department of Medical Epidemiology and Biostatistics, Karolinska Institutet, Solna, Sweden^ [↑](#footnote-ref-6)
